# Surgical Strategies for Tumors of the Pancreas and Duodenum

**DOI:** 10.3390/cancers17183091

**Published:** 2025-09-22

**Authors:** Rosyli F. Reveron-Thornton, Kelly X. Huang, Daniel Delitto, Michael T. Longaker, Jeffrey A. Norton

**Affiliations:** 1 Department of Surgery, School of Medicine, Stanford University, Stanford, CA 94305, USA; rosyrt@stanford.edu (R.F.R.-T.);; 2 Hagey Laboratory for Pediatric Regenerative Medicine, Division of Plastic and Reconstructive Surgery, School of Medicine, Stanford University, Stanford, CA 94305, USA; kellyxh@stanford.edu; 3 School of Medicine, Stanford University, Stanford, CA 94305, USA

**Keywords:** pancreatic and duodenal carcinoma, neuroendocrine tumors (pNETs), MEN-1, gastrinoma, insulinoma, pancreatic cystic neoplasms

## Abstract

The recommended surgery for pancreatic tumors is dependent on the diagnosis. For pancreatic adenocarcinoma, duodenal, and ampullary adenocarcinoma, a Whipple pancreaticoduodenectomy with lymph node dissection is recommended. For small < 2 cm or non-imageable gastrinomas, duodenal transillumination, duodenotomy, duodenal tumor excision and adjacent lymphadenectomy is recommended. For large > 3 cm gastrinomas, a Whipple pancreaticoduodenectomy with adjacent lymph node dissection is recommended. For small 1–2 cm insulinomas, intraoperative ultrasound with enucleation is recommended. If the patient with gastrinoma, insulinoma, or multiple nonfunctional NETs occurs in the setting of MEN-1, a subtotal pancreatectomy with or without splenectomy with enucleation of pancreatic head tumors is recommended, with adjacent lymph node dissection. The detail of each procedure is described with illustrations.

## Simple Summary

Tumors of the pancreas and surrounding regions are a diverse group, ranging from benign cysts to aggressive cancers. Pancreatic ductal adenocarcinoma is the most common and carries a poor prognosis, with surgery offering the only chance for cure in selected patients. Pancreatic cystic tumors, such as intraductal papillary mucinous and mucinous cystic neoplasms, require careful evaluation to determine which of these tumors require surgery and which can be safely observed. Neuroendocrine tumors vary widely, with some producing hormones and others found incidentally, and management depends on size, growth, and malignant potential. While minimally invasive methods are expanding, open surgery continues to play an important role in complex cases and surgical training. This review highlights how tumor type, location, and patient factors guide individualized treatment strategies.

## 1. Introduction

Tumors arising in the head of the pancreas represent a diverse spectrum of benign, borderline, and malignant neoplasms, each with distinct biological behavior and therapeutic implications. Accurate diagnosis, which may include pancreatic ductal adenocarcinoma, neuroendocrine tumors, and intraductal papillary mucinous neoplasms, is essential for guiding surgical decision-making. As pancreatic surgery becomes increasingly complex, choosing the optimal operative approach, whether a classic pancreaticoduodenectomy, a pylorus-preserving resection, or a minimally invasive technique, requires careful consideration of tumor type, location, and extent. Although minimally invasive methods are expanding, open approaches remain critical for complex cases and continue to play an important role in surgical training. This review highlights how tumor characteristics inform tailored surgical strategies, with particular emphasis on the role of open techniques in optimizing patient outcomes.

## 2. Pancreatic Adenocarcinoma (PDAC)

**Figure 1:**
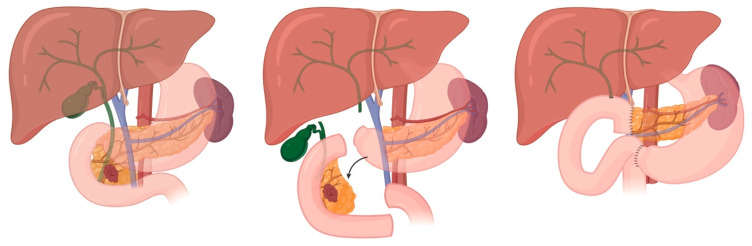
Whipple (pancreaticoduodenectomy) Overview.

PDAC is the most common pancreatic malignancy (over 90% of cases) and typically presents between the sixth and eighth decades of life, with a slight male predominance. Despite improvements in perioperative care and systemic therapy, the overall five-year survival rate remains at approximately 10%, even among those undergoing curative-intent resection [1]. While surgery plus systemic therapy remains the only potentially curative treatment, the tumor’s aggressive biology (early perineural and vascular invasion, local tissue infiltration, and dense desmoplastic stroma) poses significant challenges during resection. Only 15–20% of patients present with resectable disease at diagnosis; therefore, surgical resection with negative margins (R0 resection) and adequate lymphadenectomy is critical for long-term survival [2]. Prognostic indicators include tumor size, nodal status, resection margin, histologic grade, and preoperative serum CA19-9 levels. Increasingly, detailed vascular involvement, such as abutment or encasement of the superior mesenteric vessels (SMA and SMV), along with biologic markers, are used to guide treatment planning, including consideration of neoadjuvant therapy versus upfront surgery. Upfront surgery is currently indicated in patients with good surgical fitness, minimal to no contact with peripancreatic vasculature, and no evidence of metastatic disease [3] (Figure 1).

## 3. Pancreatic Cystic Neoplasms (PCN)

PCNs encompass a diverse group of lesions and are estimated to present in 2–45% of the general population [4,5,6,7]. This group of neoplasms includes intraductal papillary mucinous neoplasms (IPMN), mucinous cystic neoplasms (MCN), serous cystic neoplasms (SCN) and solid pseudopapillary neoplasms (SPN), each with distinct malignant potential.

SCNs are benign entities; the specific mortality due to SCNs is nearly zero [8,9]. SPNs, once considered benign, are now classified as low-grade malignant tumors, with a metastasis rate of 10–15% [10]. They predominantly affect women, with some researchers suggesting a potential hormonal influence on tumor development. MCNs are tumors with malignant potential (4–12%), typically arising in the body or tail of the pancreas in middle-aged women [11]. IPMNs are more a complex class of PCNs. They are classified into main-duct (MD-IPMN), branch-duct (BD-IPMN), and mixed-type (MT) IPMN, with the latter exhibiting features of both MD and BD subtypes. Among these, MD-IPMN carries the highest risk of progression to high-grade dysplasia (HGD) and invasive carcinoma (IC) [12]. A BD-IPMN is defined as a pancreatic cyst > 5 mm that communicates with the main pancreatic duct, whereas a MD-IPMN is characterized by diffuse or segmental dilation of the main pancreatic duct > 5 mm.

Most PCNs, apart from SPNs, are discovered incidentally. When symptoms occur, they are often nonspecific and may include abdominal or back pain, nausea, or vomiting, sometimes in the setting of pancreatitis. Other presentations include biliary obstruction caused by extrinsic compression or mucin-related blockage of the main pancreatic duct. Contrast-enhanced magnetic resonance imaging with cholangiopancreatography (MRI/MRCP) and computed tomography (CT), together with endoscopic ultrasound (EUS) and FNA-based cyst fluid analysis, are critical for distinguishing between pancreatic cyst types, stratifying the risk of high-grade dysplasia or malignancy, and informing management decisions. The literature highlights two key points regarding these diagnostic modalities. First, MRI/MRCP, CT, and EUS demonstrate comparable accuracy in diagnosing IPMNs with HGD/IC [13]. For PCNs broadly, although MRI demonstrates increased sensitivity compared to CT, the accuracy of MRI/MRCP remains relatively low (13.5–45%) so a combination of these modalities should be utilized for small PCNs or unclear diagnosis [5,14,15]. Second, the diagnostic sensitivity of cyst fluid cytology obtained via EUS-FNA is limited, with reported rates around 28.7% (range 4.8–61.5%) [16]. Therefore, a negative cytology result does not exclude the presence of HGD/IC in IPMNs. Accurate preoperative risk stratification is critical to avoid overtreatment of benign lesions while ensuring timely intervention for those with malignant potential.

**Figure 2:**
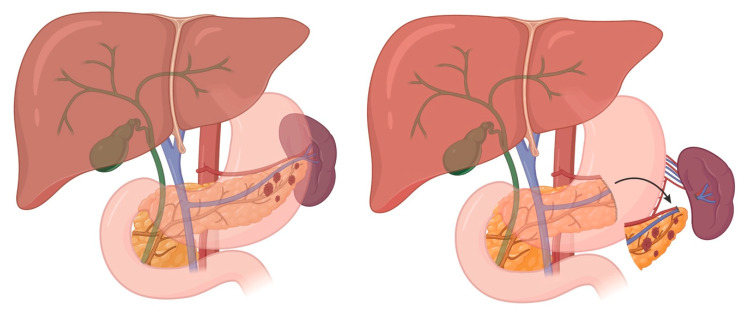
Distal Pancreatectomy Overview (tumor with adjacent lymph nodes).

**Figure 3:**
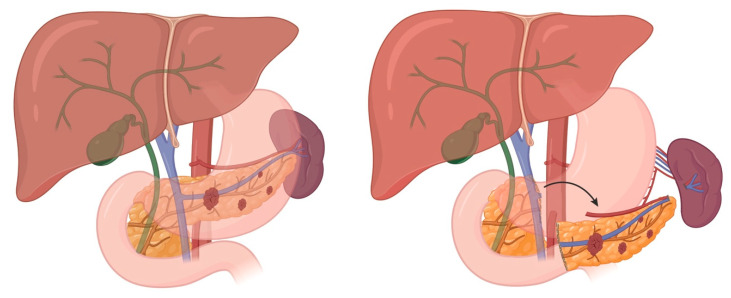
(Compare to Figure 2). Subtotal Pancreatectomy Overview (margin of resection is the SMV).

Surgical indications for PCNs are summarized in Table 1. For IPMNs, surgical candidacy is determined by the presence of “high-risk stigmata” (absolute indications), the number of “worrisome features” (relative indications), the patient’s surgical fitness, and the impact of acute pancreatitis episodes on quality of life. Generally, if invasive carcinoma is suspected, a radical pancreatectomy with lymphadenectomy should be performed; however, if the lesion is noninvasive, an organ-preserving pancreatectomy may be considered (Figure 1, Figure 2 and Figure 3). Multifocal BD-IPMNs management should be determined by the lesion having the highest risk [17].

## 4. Pancreatic Neuroendocrine Tumors (pNETs)

PNETs arise from islet cells and account for approximately 1–2% of all pancreatic cancers [20,21]. They are most often sporadic, occur more commonly in men, and are typically diagnosed between the third and sixth decades of life. Roughly 65–85% are nonfunctioning (nf-pNETs), while fewer than 20% secrete active peptides (insulin, gastrin, glucagon, VIP, or somatostatin) and are therefore classified as functional pNETs (f-pNETs) [20]. Nf-pNETs are generally larger, exhibit more heterogeneous features (such as cystic changes, calcifications, and necrosis), have a higher malignant potential (80–100%), and tend to behave more aggressively [22]. See Table 2 for pNET specific characteristics. The five-year overall survival rate for grade 1 and grade 2 pNETs is 85% and 78%, respectively [23].

The majority of pNET patients are asymptomatic; lesions are typically detected incidentally during cross-sectional imaging. Triphasic CT, MRI, EUS, and nuclear medicine imaging (such as somatostatin receptor scintigraphy) are used individually or in combination to aid in staging. Arterial calcium stimulation testing is reserved for cases of occult insulinomas, due to its invasive nature.

While surgery remains the only curative option, only 20–40% of pNETs are diagnosed at an early stage [24]. Management should be guided by a multidisciplinary team considering tumor functionality, location, size, grade, and the presence of nodal or distant metastases. It is important to note that the prognosis of pNETs vary by differentiation grade and mitotic index/Ki-67, which stratifies pNETs into three categories (G1, G2 and G3). G1 are well differentiated pNETs, characterized by <2 mitoses per 10 high-power fields (HPF) and a Ki67 index < 2%; G2 pNETs are well differentiated and defined by 2–20 mitoses per 10 HPF or a Ki67 index of 2–20%; and G3 pNETs are poorly differentiated with >20 mitoses per 10 HPF or a Ki67 index > 20%. The WHO 2017 classification further stratifies G3 pNETs into well differentiated and poorly differentiated (referred to as pNET carcinomas) [25]. Apart from G3 pNETs and patients with extrahepatic disease, surgery is indicated in all functional pNETs and nf-PNETs > 20 mm [20,26]. The role of surgery in G3 pNETs remains controversial [27,28].

**Figure 4:**
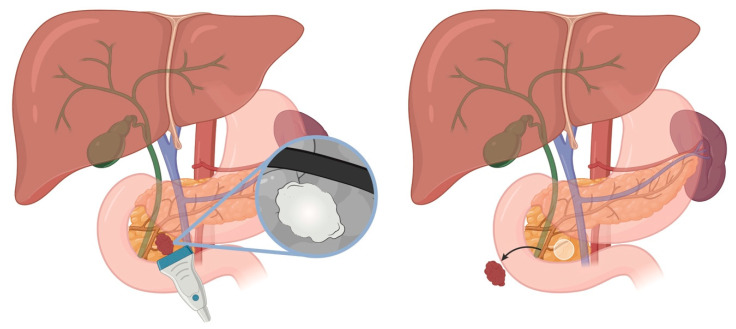
Intraoperative ultrasound identification of Insulinoma (sonolucent tumor within pancreas).

Given the low risk of malignancy, insulinomas may be managed with enucleation if located at least 3 mm from the main pancreatic duct, thereby reducing the risk of duct injury and postoperative leak (Figure 4). When enucleation is not feasible, formal oncologic resection is determined by tumor location: pancreaticoduodenectomy for tumors in the head, and distal pancreatectomy (with or without splenectomy) for those in the distal body or tail. Validated prognostic factors of metastatic relapse in resectable tumors include size > 5 cm, positive lymph nodes, Ki67 proliferative index (8–20%), and tumor grade (G3, or Ki-67 > 20%) [29]. Surveillance is preferred for small (<20 mm) nf-pNETs. We discuss management strategies for patients with Multiple Endocrine Neoplasia Syndrome-type 1 (MEN-1) below.

## 5. Sporadic Neuroendocrine Tumors of the Duodenum

**Figure 5:**
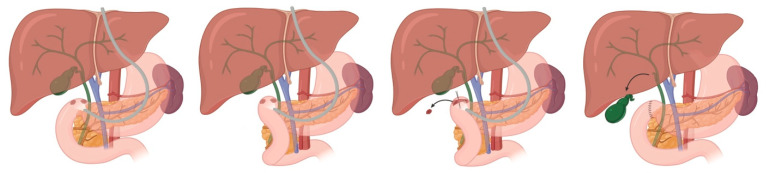
Duodenal Transillumination Overview (exposure of dNETs for resection).

Sporadic duodenal NETs (dNETs) are rare but clinically significant lesions that include gastrinoma, nonfunctioning NET, and less commonly, serotonin-producing tumors. These tumors most often occur sporadically but can be associated with genetic syndromes such as Zollinger–Ellison syndrome (ZES), multiple endocrine neoplasia type 1 (MEN1), and neurofibromatosis type 1 (NF1). DNETs can vary in behavior, ranging from indolent to aggressive, and are often discovered incidentally or during evaluation for hormone-related symptoms. Superficial, low-grade dNETs can be effectively managed with transduodenal illumination and local resection (Figure 5). This operative technique detected 75% of duodenal gastrinomas smaller than 1 cm and 83% of all duodenal gastrinomas, and was associated with improved short-term and long-term cure rates in patients with sporadic ZES [30,31]. DNETs exceeding these criteria require more formal resections that include a lymphadenectomy (Figure 1, Figure 2, Figure 3 and Figure 4).

## 6. Duodenal and Ampullary Adenocarcinoma

Duodenal and ampullary carcinomas are rare malignancies arising from the ampulla of Vater and (typically) the second portion of the duodenum. These tumors often present earlier than pancreatic cancer due to their tendency to cause biliary obstruction, leading to symptoms such as jaundice, pruritus, or cholangitis. Ampullary carcinoma generally has a more favorable prognosis than pancreatic adenocarcinoma, with five-year survival rates ranging from 30 to 50% following curative resection. Duodenal carcinoma has slightly more variable outcomes, depending on stage and histology. Both cancers require cross-sectional imaging and endoscopic ultrasound for accurate staging. For resectable disease, a pancreaticoduodenectomy (Figure 1) with en bloc lymphadenectomy remains the cornerstone of curative treatment [32]. Key prognostic factors include nodal involvement, margin status, tumor size, and histologic subtype (intestinal vs. pancreaticobiliary).

## 7. Multiple Endocrine Neoplasia-Type 1 (MEN1)

MEN1 is an autosomal dominant disorder caused by mutations in the MEN1 gene, leading to the development of various tumors including neuroendocrine tumors of the parathyroid, pituitary, and pancreas. A total of 20 to 80% of MEN1 patients develop f-pNETs and 80–100% develop nf-pNETs [33]. Furthermore, 30–90% of MEN1 patients develop both duodenal and pancreatic neuroendocrine tumors (dpNETs) [34,35]. Although the most common manifestation of the disease is hyperparathyroidism, up to 33% present with f-pNET [33]. This is significant because pNETs are the usual cause of death in MEN1 patients [36]. The most common f-pNET in MEN1 patients is insulinoma (30% of cases) and gastrinoma (most commonly in duodenum, <10% are pancreatic) [35]. Studies suggest that pNETs > 2 cm and patients > 40 years of age are both associated with an increased risk of developing distant metastases and mortality [37].

This nature of this disease presents many therapeutic challenges. For example, nf-pNETs localized to the pancreas are often microscopic and multifocal with only a small percentage of patients becoming symptomatic. A total of 85–100% of gastrinomas in MEN1 patients with ZES occur multifocally in the duodenum, while the remaining 15% are dpNETs. Additionally, these gastrinomas are frequently small (<0.5 cm) and associated with lymph node metastases in 40 to 60% of patients [33]. Incomplete tumor resection and multifocality may result in persistent tumor and subsequent local recurrence.

Both domestic [26,35] and international consensus statements [38,39] recommend active surveillance for MEN1 patients with ZES or nf-pNETs tumors < 1–2 cm, as studies did not find a significant benefit of surgery in these patients [40]. Surgical intervention is warranted for all functional tumors and nf-pNETs > 2–3 cm or those that show signs of progression on imaging studies during observation. Due to the multifocal nature of insulinomas in MEN-1 patients, a calcium arteriogram is often helpful in identifying the precise region of the pancreas affected [41,42].

**Figure 6:**
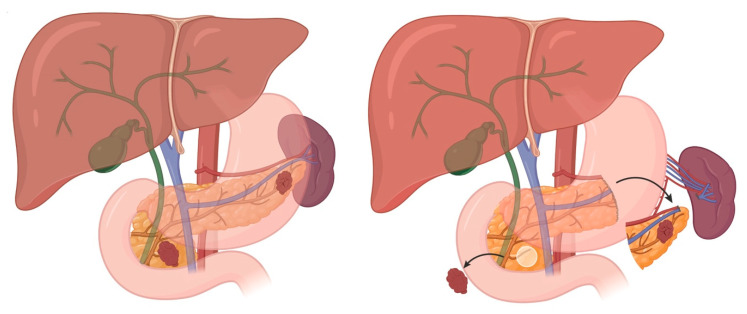
Approach to MEN1 Overview.

It is now generally recognized that these patients cannot be completely cured of all disease without aggressive surgery. However, the current literature does not establish whether performing an extensive partial pancreatectomy reduces the risk of malignant progression or the development of new tumors with malignant potential in the remaining pancreatic tissue [43,44,45]. Therefore, the type and extent of pancreatic surgery must be individualized with considerations of age at presentation, tumor size, growth rate, and location to try and minimize long-term complications (Figure 6).

## 8. Surgical Considerations

### 8.1. Open vs. Minimally Invasive

The surgical approach is typically determined by preoperative imaging and anticipated intraoperative complexity. Open pancreaticoduodenectomy (Whipple) is most commonly performed through a midline laparotomy or bilateral subcostal (Chevron) incision to facilitate the exposure of the pancreatic head, the uncinate process, and the surrounding vasculature. Similarly, open sub-total and distal pancreatectomies are approached via a midline or left subcostal incision. Minimally invasive techniques, including laparoscopic and robotic surgery, utilize four to six trocar sites across the upper abdomen. For laparoscopic cases, a 10–12 mm camera port is typically placed at or above the umbilicus, with additional working ports in the bilateral upper quadrants. Robotic resections (e.g., using the da Vinci system) follow a similar configuration, with 8 mm ports for the robotic arms and a 12 mm assistant port for suction, stapling, and specimen retrieval.

Compared with open surgery, minimally invasive approaches are associated with reduced intraoperative blood loss, lower wound-related morbidity, shorter length of stay, and faster recovery [46,47,48]. However, open surgery remains the preferred approach in complex scenarios, including locally advanced tumors requiring complex vascular resection/reconstruction, medically complex patient, dense adhesions from multiple prior laparotomies, or extensive retroperitoneal invasion. For pancreaticoduodenectomy, large database studies and randomized trials suggest that minimally invasive techniques can achieve comparable oncologic outcomes in high-volume centers, but these procedures are associated with longer operative times and require significant technical expertise [49,50,51,52,53]. Therefore, contraindications for a minimally invasive procedure includes inability to tolerate pneumoperitoneum, untreated coagulopathy or liver failure, documentation of hostile abdomen, vascular involvement, invasion into the retroperitoneum, and sequelae of acute/chronic pancreatitis or radiation that drastically increases the risk of complications [54].

### 8.2. Pancreaticoduodenectomy (Whipple Procedure)

#### 8.2.1. Indications

- PDAC, nf-pNET > 2 cm, node-positive tumors, and f-pNET (insulinoma can be enucleated if located ≥3 mm from the main pancreatic duct).
- IPMN with “high-risk stigmata” or cyst with high-risk features concerning for HGD or IC.
- Malignant or high-risk lesions of the distal bile duct and ampulla of Vater.
- Consider for all duodenal malignancies, particularly if located in the second portion of duodenum, or if the tumor invades any portion of ampulla or pancreas.

#### 8.2.2. Preoperative

- It is important to identify any aberrant vascular anatomy (especially the replaced right hepatic artery) prior to start of the case, as damage to these vessels can unexpectedly compromise blood flow to the liver, intestines, and spleen.
- If available, an epidural serves as an important option for pain control.
- The patient is placed in a supine position with both arms tucked. A foley catheter, arterial line, and nasogastric tubes are placed, followed by the induction of general anesthesia.
- Antibiotic coverage should include drugs for gram-positive skin flora, gram-negative, and anaerobic intestinal bacteria.

#### 8.2.3. Steps of the Procedure [55]

After gaining access to the abdominal cavity via an open or minimally invasive approach, a thorough inspection of the abdominal contents and viscera is performed to assess for any evidence of metastatic disease. First, a Cattell-Braasch maneuver (right to left medial visceral rotation) is performed to expose the second and third portions of the duodenum and the pancreatic head. A Kocher maneuver is performed, beginning at the lateral edge of the duodenum and extending cephalad toward the foramen of Winslow and caudally to the left renal vein after it has crossed over the aorta. It is essential to continue dissection to the left renal vein, as this plane contains an avascular ligament that tethers the third portion of the duodenum inferiorly, preventing complete mobilization of the duodenum and pancreas from the inferior vena cava and aorta. The gastrocolic ligament is then divided outside of the gastro epiploic vessels to open the lesser sac and expose the body and tail of the pancreas while preserving blood supply to the duodenal cuff and stomach. Attachments from the posterior stomach to the anterior aspect of the pancreatic body are taken down for full exposure of the pancreas. The middle colic and gastroepiploic vein are identified as they enter the SMV anteriorly, and dissected circumferentially, before being ligated and divided. The inferior border of the pancreas is further dissected to identify it and expose the SMV. After completing pancreatic exposure, dissection proceeds at the porta hepatis to expose the common hepatic artery and its branches. Once aberrant anatomy is excluded, the common hepatic artery is exposed. This can be accomplished by removing the hepatic artery node sitting anterior to the vessel and ligation of the right gastric artery. Adequate exposure of the common hepatic artery allows the identification of the gastroduodenal artery (GDA) arising from the posterior aspect of the hepatic artery. The GDA is dissected and test clamped with a bulldog to ensure sufficient blood flow through the proper hepatic artery. If adequate, the GDA is ligated and divided using a vascular stapler, allowing medial and cephalad retraction of the hepatic artery and its branches to expose the supra-pancreatic aspect of the portal vein. A tunnel is then created between the pancreatic neck and the inferior portal vein. The common bile duct, cystic duct, and cystic artery are dissected andligated prior to cholecystectomy.

Once all the major structures have been dissected, the structures to be used in the reconstruction are divided. The duodenum is divided approximately 2 cm distal to the pylorus (pylorus-sparing Whipple), or alternatively for a proximal duodenal tumor the antrum is divided. The jejunum is then divided, 10 cm distal to the ligament of Treitz. The common bile duct is sharply divided, proximally to the cystic duct. The neck of the pancreas is suture ligated at the superior and inferior border then transected at the start of the portal vein. The uncinate process is then dissected from the superior mesenteric vessels. Once the specimen is removed en bloc, reconstruction is performed using three anastomoses: the pancreatojejunostomy (PJ), hepaticojejunostomy (HJ), and either a gastrojejunostomy (DJ) or duodenojejunostomy (DJ), depending on the approach. Meticulous attention to hemostasis, anastomotic technique, and drain placement is critical to minimize postoperative complications such as fistula, hemorrhage, or delayed gastric emptying.

#### 8.2.4. Complications

- Delayed gastric emptying.
- Pancreatic leak.
- Pancreatic fistula.
- Pseudo aneurysm.
- Wound infection.
- Abdominal abscess.

According to the ISGPS [56], a postoperative biochemical leak is defined as drain fluid with amylase >3 times the upper limit of normal, without clinical impact. A clinically relevant postoperative pancreatic fistula is defined as grade B, requiring a change in management such as prolonged drainage (>3 weeks) or interventional procedures, and grade C, which is associated with organ failure, reoperation, or mortality. Management ranges from conservative therapy (prolonged drainage, antibiotics, and/or nutritional support) to percutaneous/endoscopic drainage, with surgery reserved for severe cases. Delayed gastric emptying is similarly graded (A–C) by ISGPS, based on the need for nasogastric decompression and delayed oral intake, with treatment focused on supportive care, prokinetics, and nutritional support [57].

### 8.3. Subtotal Pancreatectomy with or Without Splenectomy, Distal Pancreatectomy with or Without Splenectomy (Lateral to the Superior Mesenteric Vessels)

#### 8.3.1. Indications

- Non-spleen preserving: PDAC, nf-pNET > 2 cm, node-positive tumors, and f-pNET
- Spleen preserving: Patients with low-risk sporadic pNETs, such as small, well differentiated or cystic lesions, who are younger and have an expected long-term survival are ideal candidates for planned splenic preservation.

#### 8.3.2. Preoperative

- The patient is positioned supine, with left arm out and right arm tucked to allow the placement of the self-retaining retractor on the patient’s right-hand side.
- Prophylactically vaccinate patients against encapsulated bacteria (*Streptococcus pneumonia*, *Neisseria meningitidis*, and *Haemophilus influenza* B) two weeks prior to elective splenectomy.
- A foley catheter, arterial line, and orogastric tubes are placed followed induction of general anesthesia.
- Antibiotic coverage should include gram-positive skin flora, gram-negative, and anaerobic intestinal bacteria.

#### 8.3.3. Steps of the Procedure

The abdomen is accessed by either a left subcostal or midline incision. The abdomen is again explored for signs of metastatic disease. Following the placement of a self-retaining retractor, the gastrocolic ligament is incised below the neck of the pancreas and taken laterally to the splenic flexure, allowing adequate access to the lesser sac and pancreas. If the spleen will not be preserved, the short gastric vessels are divided to allow for exposure of the posterior gastric attachments to the pancreas. Once divided, the stomach is retracted superiorly to expose the anterior surface of the pancreas. The junction of the splenic vein to the portal vein is identified on the inferior aspect of the pancreas, and isolated. The same procedure is conducted for the splenic artery running superiorly to the pancreas. The decision to ligate the splenic artery and splenic vein, as well as to perform a retrograde (lateral-to-medial) versus antegrade (medial-to-lateral) dissection of the pancreas, is determined by the extent of the tumor and whether a simultaneous en bloc splenectomy is planned. See the indications for a splenectomy above.

The Warshaw technique is a popular technique for spleen preservation and involves dividing the splenic artery and vein proximal to the tumor and then again at the splenic hilum. The spleen blood flow is then supplied primarily by the short gastric vessels arising from the gastroepiploic arcade. More commonly, these splenic vessels are preserved through careful dissection of the posterior pancreas to the splenic hilum. The spleen can be transected using a thoracoabdominal stapler (TA) with a covered staple line or with an energy device with subsequent over-sewing. A tip of a 19 French, round Blake drain (Or 10 French flag JP drain) is positioned near the pancreatic stump.

#### 8.3.4. Complications

- Pancreatic fistula.
- Pancreatic leak.
- Bleeding.
- Infection.
- Pseudocyst.

### 8.4. Enucleation

#### 8.4.1. Indications

Insulinomas, gastrinomas, and nf-pNETs with a low suspicion of malignancy < 2 cm. These tumors should be >3 mm from the main pancreatic duct to minimize the risk of pancreatic fistula.

#### 8.4.2. Preoperative

- The patient is positioned supine, with left arm out and right arm tucked to allow the placement of a self-retaining retractor on the patient’s right-hand side.
- A foley catheter and orogastric tubes are placed, followed by the induction of general anesthesia.
- For gastrinoma patients, the PPI is given preoperatively and continued postoperatively.
- For insulinoma patients, glucose is infused continually until the tumor is removed to reduce the risk of hypoglycemia.

#### 8.4.3. Steps of the Procedure

An upper midline incision establishes access to the abdomen to begin our exploration for occult metastatic disease. The greater omentum is swept cranially and the gastrocolic ligament is incised to enter the lesser sac; this provides access to lesions in the head, body, and tail of the pancreas. If more mobilization is required, additional techniques include mobilization of the superior and inferior borders of the pancreas, as well as tunneling behind the neck of the pancreas and the portal vein to perform a hanging maneuver to give the surgeon more traction on the pancreas. Intraoperative ultrasound (IOUS) can then be used to localize occult tumors and confirm adequate distance from the pancreatic duct. The original studies advocating for the use of IOUS [58,59,60] demonstrated that 66% of the insulinomas in the pancreatic head were nonpalpable, advocating for the standard use of IOUS in all cases. Once resectability is confirmed, the tumor is dissected from the pancreatic parenchyma using blunt dissection along the periphery of the tumor with blood vessel bleeding control with the Ligasure or the harmonic scalpel. IOUS can be used to be certain that the tumor is completely excised. When using these techniques in insulinoma or gastrinoma, there is a very low probability of incomplete resection and recurrence.

#### 8.4.4. Complications

- Pancreatic fistula.
- Acute pancreatitis.
- Hemorrhage.
- Pseudocyst.
- Infection.
- Disease recurrence.

### 8.5. Duodenal Transillumination and Resection

#### 8.5.1. Indications

Nf-NETs, gastrinomas, somatostatinomas that are <1–2 cm in size, confined to the submucosa (T1 lesions), well differentiated (Grade 1 or low Grade 2), with or without evidence of lymph node metastasis.

#### 8.5.2. Preoperative

- For gastrinoma patients, a proton pump inhibitor (PPI; 40 mg omeprazole) is given preoperatively and continued postoperatively every 12 h.

#### 8.5.3. Steps of the Procedure

In the ZES patients, a proton pump inhibitor is given preoperatively and continued throughout the procedure (usually twice the normal dose of iv PPI every 12 h)**.** The abdomen is accessed by an upper midline incision. An extended Kocher maneuver is performed to mobilize the duodenum and pancreas sufficiently to allow circumferential palpation. The duodenum and the pancreas are then examined using an intraoperative ultrasound, as previously described. An endoscope is then passed orally into the duodenum; the operating room lights are dimmed. From the pylorus to the ligament of Treitz, the duodenum is examined by transillumination from the serosal side by the surgeon, while simultaneously being examined endoscopically from the luminal side by the endoscopist [58]. The tumor will be seen as an opaque mass during the transillumination. If the tumor is on the medial wall near the pancreas, a cholecystectomy is performed to allow placement of a Fogarty catheter with a balloon to help identify the ampulla on the medial wall of the duodenum. Once the location of tumors and number of tumors is confirmed, electrocautery is used to create a longitudinal duodenotomy. If near the pylorus, it may be included in the incision. Two 3–0 sutures placed on the incisional flaps at the level of the tumor facilitate visualization and dissection. It is important to remember that the ampulla is usually located at the junction of the lower third with the upper two-thirds of the second portion of the duodenum to prevent inadvertent injury. The lesion is circumferentially resected under visual guidance and palpation, with and without the aid of frozen sections of the margin. The duodenum is repaired transversely using a layer of full-thickness PDS, reinforced with interrupted seromuscular silk suture to avoid duodenal stenosis and leaks. Esophagogastroduodenoscopy is utilized as needed to assess for leak and lumen patency. For patients with ZES, the proton pump inhibitor (PPI) is continued postoperatively and in the hospital and for three months, as acid secretion does not return to normal until the gastric wall hypertrophy resolves.

#### 8.5.4. Complications

- Duodenal leak.
- Gastric outlet obstruction.
- Infection.
- Bleeding.
- Damage to ampulla of Vater, leading to obstructive biliary symptoms.

### 8.6. MEN-1

#### 8.6.1. Indications for Resection

Nf-pNETs > 2 cm, insulinoma, gastrinoma and other functional pNETs.

#### 8.6.2. Preoperative

- The use of glucose for insulinoma and PPI for gastrinoma preoperatively and throughout the procedure has already been described.
- MEN-1patients with ZES usually have multiple pNETs and multiple duodenal NETs.
- Since pNETs are the major determinant of survival in MEN-1 patients [36], the goal of surgery is to remove all enlarged pNETs and dNETs. This usually requires a subtotal pancreatectomy from the SMV to the tail and enucleation of remaining pNETs in the pancreatic head and excision of dNETs. These procedures are described above.

### 8.7. Lymphadenectomy

An important prognostic marker of survival and predictor of recurrence in malignant pancreatic and duodenal neuroendocrine tumors is lymph node metastasis. A 2016 consensus statement by the International Study Group on Pancreatic Surgery (ISGPS) [61] recommends a standard lymphadenectomy for pancreatoduodenectomy that includes lymph node stations 5, 6, 8a, 12b1, 12b2, 12c, 13a, 13b, 14a, 14b, 17a, and 17b. The standard lymphadenectomy for tumors of the pancreatic body and tail include lymph node stations 10, 11, and 18. A study by Malleo et al. [62] reassesses the optimal number of examined LNs in patients undergoing Whipple procedures for PDAC and found that examining at least 28 LNs ensured optimal staging through improved detection of N2/stage 3 disease [62]. Extended lymphadenectomies for PDAC management were first described in 1973, but its use continues to be heavily debated due to a lack of survival advantages in various randomized control trials [63,64,65] and meta-analyses [66,67,68].

## Figures and Tables

**Table 1:** Indications for Surgery in Pancreatic Cystic Neoplasms (PCNs).

**IPMN** [17,18,19]	“High risk stigmata”/Absolute indications [17,18,19] o Obstructive jaundice in a patient with a cystic lesion located in the pancreatic head o Enhancing mural module ≥ 5 mm or containing a solid component o MPD ≥ 10 mm o Positive (HGD/IC) or suspicious cytology Surgically fit patients with MD-IPMN (GRADE 1B, strong agreement) or MT-IPMN (GRADE 2C, strong agreement) [19] Refer to Kyoto Guidelines [17] and The European Study Group on Cystic Tumors of the Pancreas Guidelines [19] for “worrisome features” and relative indications for surgery 9 September 2025 10:24:00 AM
**MCN** [19] (Grade 1B, strong agreement)	Size ≥ 40 mm Symptomatic Risk factors present including mural nodules **or** solid components Cytology shows atypia or high-grade dysplasia Rate of size increase, particularly during pregnancy (relative indication)
**SCN** [19] (Grade 2C, strong agreement)	Symptomatic
**SPN** [19] (Grade 1B, strong agreement)	All SPNs should undergo radical resection due to uncertainty of malignant potential

Intraductal Papillary Mucinous Neoplasm (IPMN), Mucinous Cystic Neoplasm (MCN), Serous Cystic Neoplasm (SCN), Solid Pseudopapillary Neoplasm (SPN). Main-duct IPMN (MD-IPMN); Mixed-type IPMN (MT-IPMN); Main pancreatic duct (MPD); high grade dysplasia/invasive carcinoma (HGD/IC).

**Table 2:** Pancreatic neuroendocrine tumor characteristics.

pNET	Incidence	Malignancy Risk	Location	Clinical Syndrome/Key Features
**Non-functional**	65–85%	High (~60–90%)	Head (~40–60%) or body	Asymptomatic; may present with mass effect symptoms (e.g., pain, weight loss)
**Insulinoma**	10–20%	Low (~5–10%)	Head = body = tail solitary, lesions	Hypoglycemia, neuroglycopenic symptoms
**Gastrinoma**	5–10%	Moderate to High (~60%)	Duodenum (most common), pancreatic head (Gastrinoma Triangle)	Zollinger-Ellison syndrome (peptic ulcers, acid hypersecretion)
**Glucagonoma**	<5%	High (>70%)	Pancreatic tail	Necrolytic migratory erythema, diabetes, weight loss
**VIPoma**	<2%	High (~60%)	Pancreatic tail	WDHA syndrome (watery diarrhea, hypokalemia, achlorhydria)
**Somatostatinoma**	<1%	High (>70%)	Pancreatic head or duodenum	Diabetes, steatorrhea, gallstones

## Data Availability

No new data were created or analyzed in this study.
